# Supplementary material for: Quorum sensing regulates virulence factors in the coral pathogen Vibrio coralliilyticus
Source: Appl Environ Microbiol. 2025 Jan 15;91(2):e01143-24. doi: 10.1128/aem.01143-24 (PMC11837519; doi:10.1128/aem.01143-24)
Supplement: Supplemental material — Figures S1 to S5; Tables S1 to S3. [file aem.01143-24-s0001.pdf]

Quorum Sensing Regulates Virulence Factors in the Coral Pathogen *Vibrio coralliilyticus*.

Victoria N. Lydick, Shir Mass, Robert Pepin, Ram Podicheti, Emra Klempic, Douglas B. Rusch, Blake Ushijima, Laura C. Brown, Dor Salomon, and Julia C. van Kessel

Supplemental Figures 1-5
Supplemental Tables 1-3
References

Figure S1

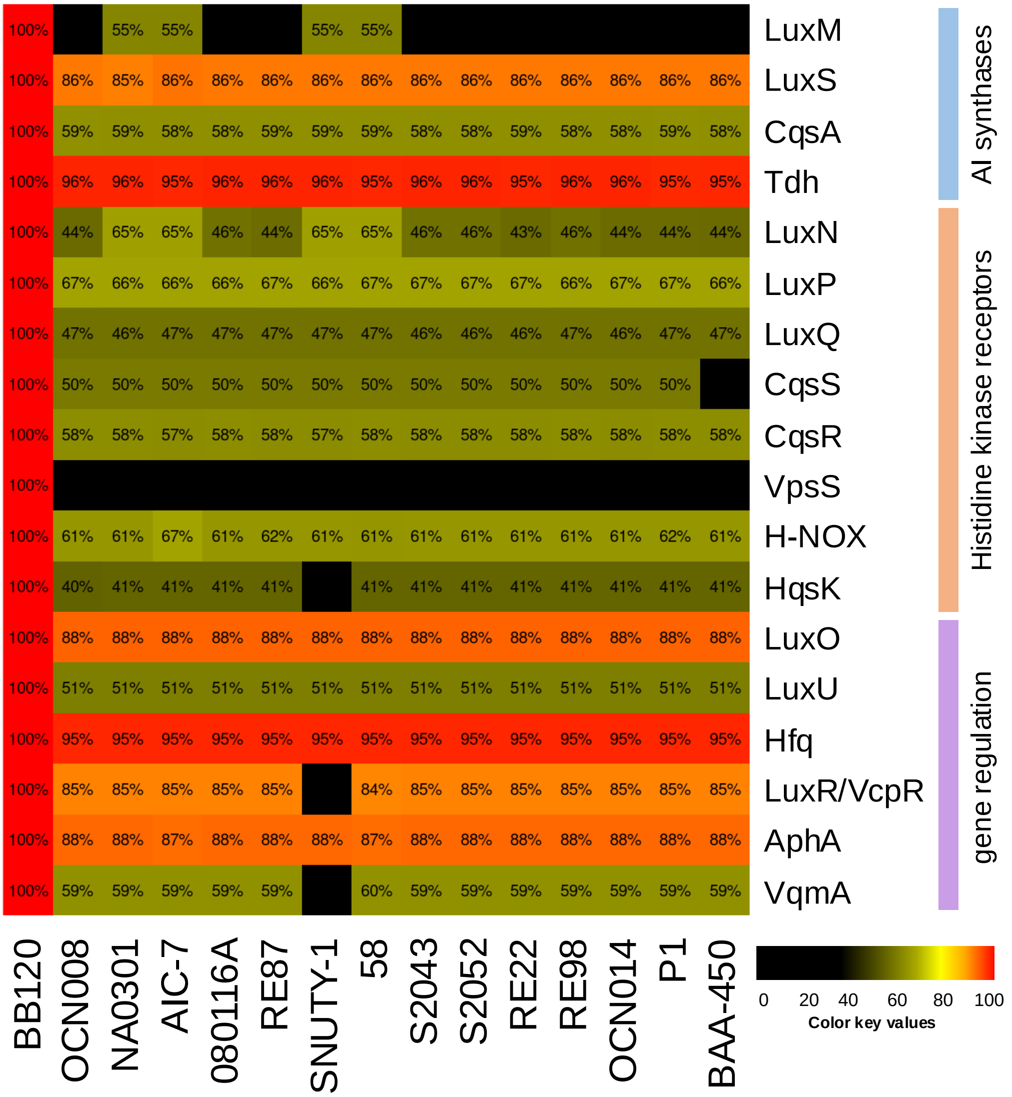

Figure S1. Conservation of QS system homologs in *Vcor* strains. The proteins listed on the y-axis are involved in QS in *V. campbellii* BB120 (query sequences) and are grouped based on function. The heat-map indicates conservation of these proteins in *Vcor* strains (amino acid identity); 40% identity or lower is colored in black.

Figure S2

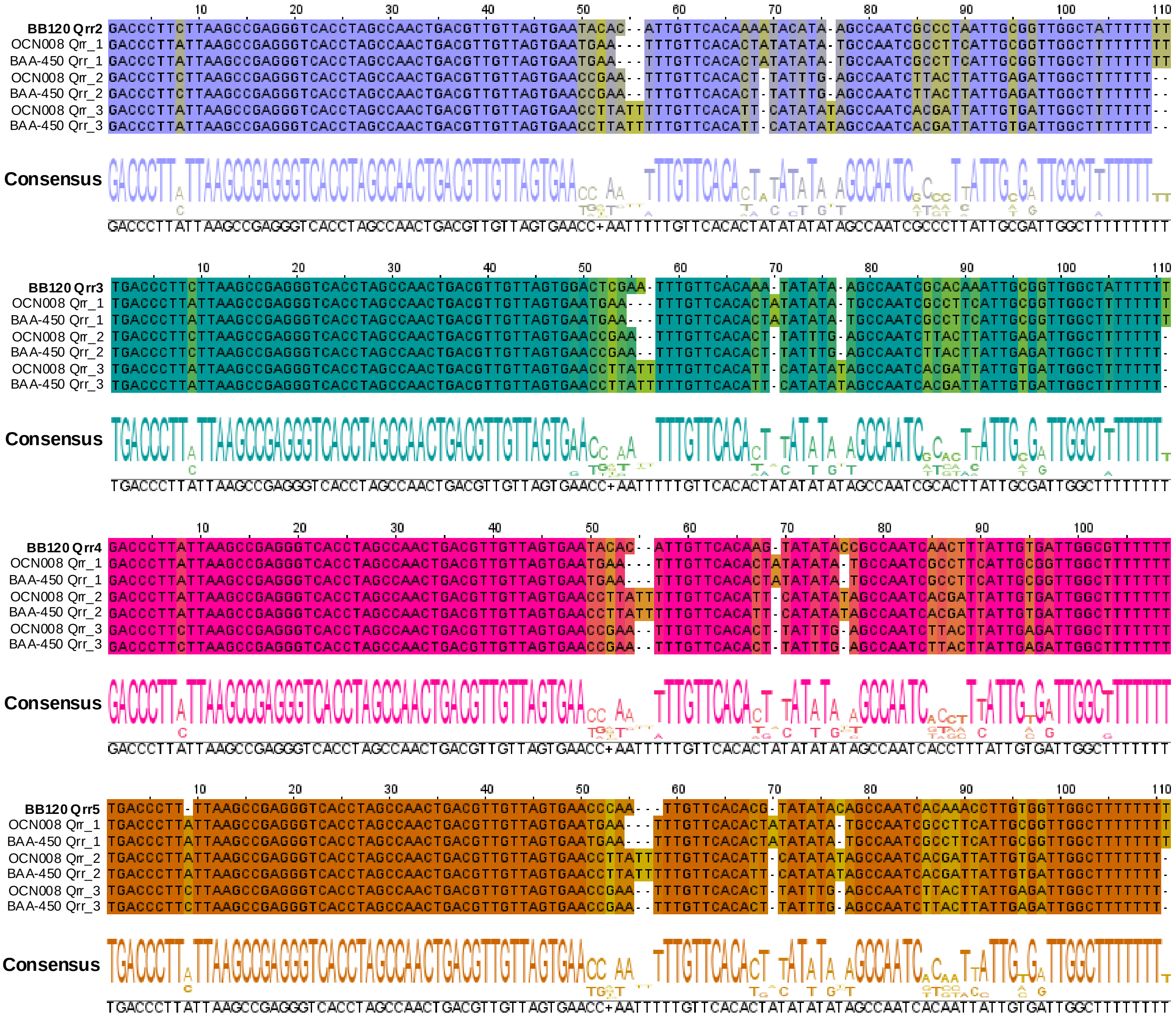

Figure S2. Sequence alignments of Qrr genes from *Vibrio campbellii* and *Vcor*. Nucleotide alignments and consensus sequences of three Qrr genes identified in *Vcor* strains OCN008 and BAA-450 with reference to *V. campbellii* BB120 Qrr genes 3-5. Alignment images created using Jalview Version 2 system.

Figure S3

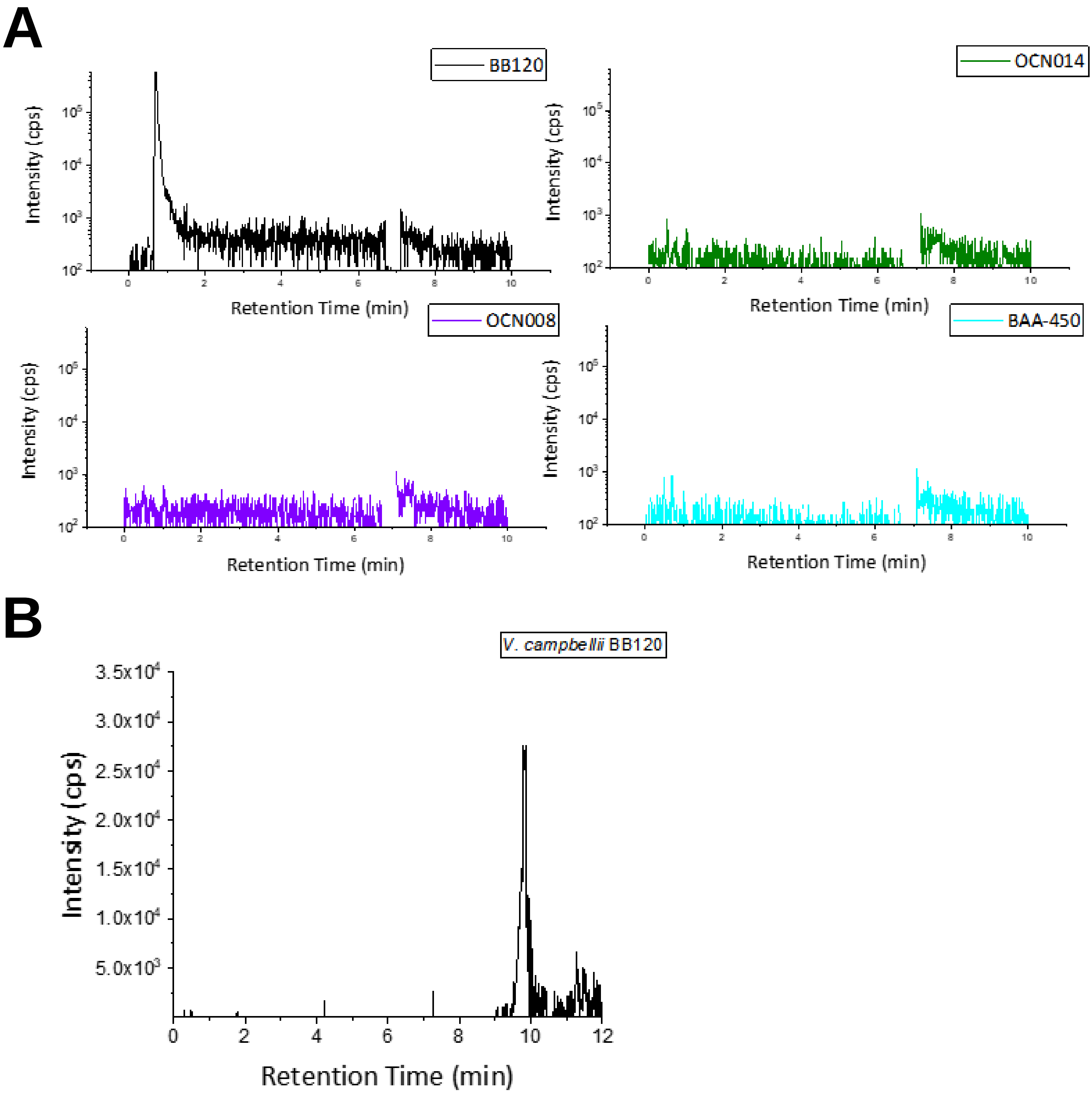

Figure S3. Detection of autoinducers using mass spectrometry. (A) Total ion chromatograms of AHL molecules in extracts of supernatant from strains *Vcor* OCN008, OCN014, and BAA-450, and positive control strain *V. campbellii* BB120. (B) Base peak chromatogram (BPC) and extracted ion chromatogram (EIC) detection of enamine CAI-1 from *V. campbellii* BB120 supernatant extract.

Figure S4

A

AI-2 Formation:

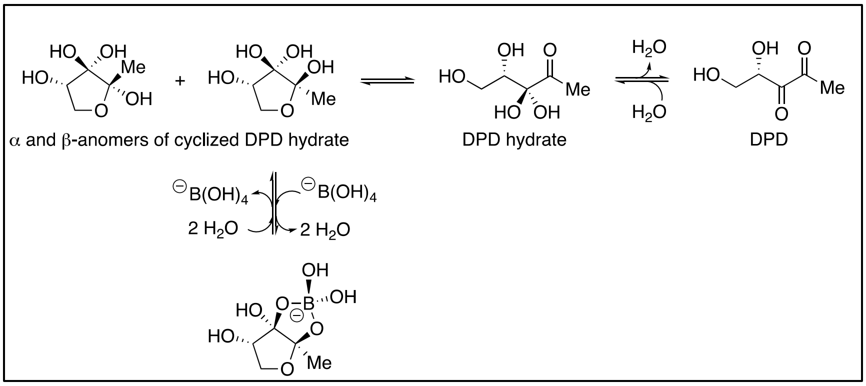

B

Derivatization and detection of DPDQ:

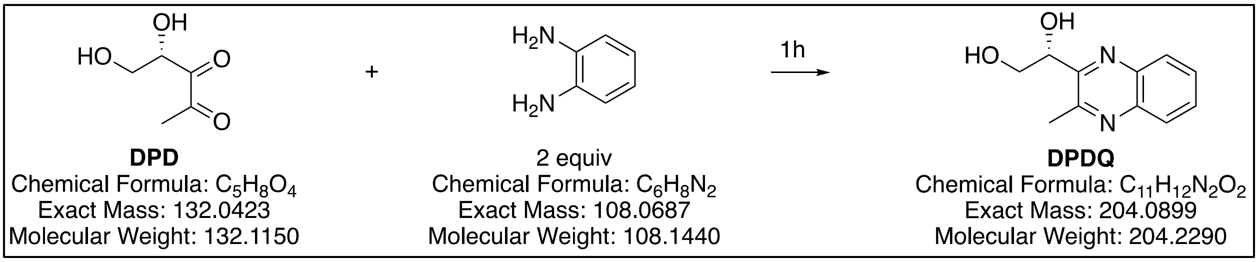

Figure S4. AI-2 and derivatize compounds detected by mass spectrometry. (A) AI-2 precursor molecule (DPD) and addition of boric acid to form AI-2. (B) DPD with the addition of chemical OPD forms the detectable compound, DPDQ.

Figure S5

A

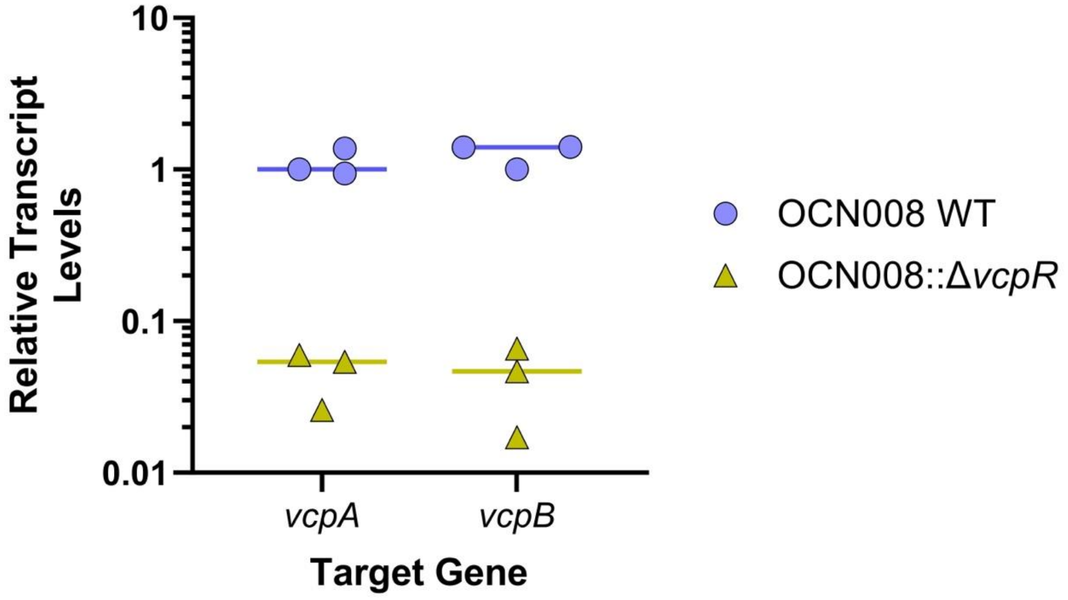

B

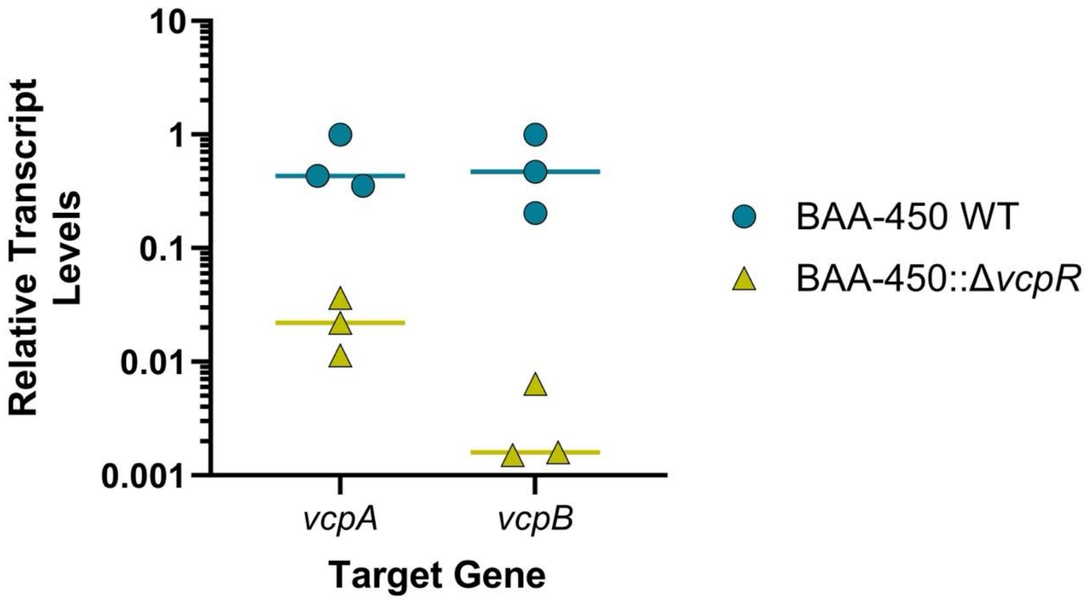

Figure S5. VcpR regulates the protease-encoding genes, *vcpA* and *vcpB*. Reverse transcriptase quantitative PCR (RT-qPCR) of transcript levels for target genes *vcpA* and *vcpB* comparing wild-type and Δ*vcpR Vcor* strains of parent OCN008 (A) and BAA-450 (B); *recA* was used as the standard control for analyses.

Table S1. Bacterial strains used in this study.

Strains		
**Name**	**Description**	**Reference**
OCN008	*V. coralliilyticus* type strain (wild type)	Ushijima et al. 2014 (1)
BAA-450	*V. coralliilyticus* type strain (wild type)	American Type Culture Collection (ATCC), atcc.org
OCN014	*V. coralliilyticus* type strain (wild type)	Ushijima et al., 2016 (2)
BB120	*V. campbellii* type strain (wild type)	Bassler et al., 1997 (3)
VL010	OCN008::Δ*luxR*	Guillemette et al., 2020 (4)
VL030	OCN008::Δ*luxO*	Lab Collection
VL027	OCN008::Δ*luxN*	Lab Collection
VL028	OCN008::Δ*luxP*	Lab Collection
VL029	OCN008::Δ*cqsS*	Lab Collection
VL034	OCN008::pCS18, kan^R	This study
VL035	OCN008::Δ*vcpR*, pCS18, kan^R	This study
VL044	OCN008::pCS19, kan^R	This study
VL045	OCN008::Δ*vcpR*, pCS19, kan^R	This study
VL018	BAA-450::Δ*vcpR*	This study
VL019	BAA-450::Δ*luxO*	This study
VL038	BAA-450::pCS18, kan^R	This study
VL011	OCN014::Δ*vcpR*	This study
VL036	OCN014::pCS18, kan^R	This study
VL037	OCN014::Δ*vcpR*, pCS18, kan^R	This study
KM816	BB120::Δ*luxS*	Waters and Bassler, 2006 (5)
TL203	BB120::Δ*cqsA*	Simpson et al., 2019 (6)
JMH363	BB120::Δ*luxM*	Waters and Bassler, 2006 (5)
TL189	BB120::Δ*luxS, ΔcqsA, ΔluxM*	Simpson and Petersen et al., 2021 (7)
VL066	OCN008:: Δ*hcp1*	Mass et al, 2024 (8)
VL067	OCN008:: Δ*tssM2*	Mass et al, 2024 (8)
ecVL017	*E. Coli* b3914 (wild-type)	Lab Collection

Vnat::pBAD33	*Vibrio natriegens* ATCC 14048 wild-type::pBAD33; Cm^R	Mass et al, 2024 (8)

Table S2. Plasmids used in this study.

Plasmids		
**Name**	**Description**	**Reference**
pCS18	$P_{luxCDABE}$; kan^R; derivative of pMMB67EH-tfoX-kanR	Chelsea Simpson
pCS19	$P_{luxCDABE}$::*gfp*; kan^R; derivative of pMMB67EH-tfoX-kanR	Simpson et al., 2019 (6)
pCS42	$P_{luxCDABE}$::*gfp*; $gent^R$; derivative of pMMB67EH-tfoX-gentR	Chelsea Simpson
pVND01	*E. Coli* b3814::pSW4426T; Cm^R, Sp^R, Sm^R, R6k oriV, PBAD-ccdB; DAP auxotroph; suicide vector to delete LuxO in *Vcor* ATCC BAA-450	This study
pVND02	*E. Coli* b3814::pSW4426T; Cm^R, Sp^R, Sm^R, R6k oriV, PBAD-ccdB; DAP auxotroph; suicide vector to delete VcpR in *Vcor* ATCC BAA-450	This study
pBU247	*E. Coli* b3814::pBU247; Cm^R; DAP auxotroph; suicide vector to delete Tssm1 in *Vcor* OCN008	Lab Collection
pRK600	Helper plasmid	
pSW4426T	*E. Coli* b3814::pSW4426T; Cm^R, Sp^R, Sm^R, R6k oriV, PBAD-ccdB; DAP auxotroph; empty suicide vector	Le Roux et al., 2007 (9)

Table S3. Oligonucleotides used in this study.

DNA oligonucleotides			
**Primer**	**Description**	**Sequence**	**Source**
VNL019	Forward amplify pSW4426T backbone (universal)	5’-tcgcacgatatacaggattttgcc-3'	This study
VNL062	Reverse amplify pSW4426T backbone (universal)	3’-gcgaaggatctcttcagctcagtc-5'	This study
VNL058	Amplify downstream forward insert (vcpR)	5’-TATATAGAATTCgaaaactacttatcatgggcggt-3’	This study

VNL044	Amplify downstream reverse insert (vcpR)	3’-TATATACTCGAGattaaccagtgtcatcgtaaagccg-5'	This study
VNL043	Amplify upstream forward insert (vcpR)	5’-TATATACTCGAGagttatatttccttgccaattgagtt-3'	This study
VNL055	Amplify upstream reverse insert (vcpR)	3’-TATATATCTAGAtggcaagctgcaagatttag-5'	This study
VNL062	Sequencing detection primer for section between UP and DOWN arms (pVND02, vcpR)	5’-AAGGCATCAAGTACGAAGTAGC-3'	This study
VNL066	Gene locus upstream forward (vcpR BAA-450)	5’-gagtgaacgcagcaatcgacaac -3’	This study
VNL067	Gene locus downstream reverse (vcpR BAA-450)	3’-tgcgtaatgtgacggttgttaacg -5’	This study
VNL046	Amplify upstream forward insert (luxO)	5’-TATATAgaattcaggacgaacgtgtgttggtcaccac-3’	This study
VNL047	Amplify upstream reverse insert (luxO)	3’-TATATACTCGAGcaaaagatatttggacttttgtgtttgc-5’	This study
VNL048	Amplify downstream forward insert (luxO)	5’-TATATACTCGAGTCGATCATGGAAGTGTTAAATCAAAC-3’	This study
VNL057	Amplify downstream reverse insert (luxO)	3’-TATATAtctagaTTCGATGATGTTTGAATATCGCTTCA-3’	This study
VNL064	Gene locus upstream forward (luxO BAA-450)	5’-aagttcactaacaacgtcagttggc-3’	This study
VNL065	Gene locus downstream reverse (luxO BAA-450)	3’-gctgtgtcgctgagatatcgagc-5’	This study
VNL063	Sequencing detection primer for section between UP and DOWN arms (pVND01, luxO)	5’-ccgtcgtagagagaaacaacaagc-3’	This study

JCV612	Forward sequence of PluxC PCR filter binding probe	5’-cttatgaagtccatacttttcactgaa-3’	Julia van Kessel
JCV613	Reverse sequence of PluxC PCR filter binding probe	3’-tttgcccatttattattaaaggtaagtgtttt-5’	Julia van Kessel
VNL022	qRT-PCR reference gene OCN008 recA forward	5’-ATGAACAAATCGGAGAAAGTGATG-3’	This study
VNL023	qRT-PCR reference gene OCN008 and BAA-450 recA reverse	5’-gcataatagagcctttaccgaattg-3’	This study
VNL069	qRT-PCR reference gene BAA-450 recA forward	5’-ttgattgcctcagaggactc-3’	This study
VNL024	qRT-PCR OCN008 and BAA-450 vcpA forward	5’-atggttaaagttgaaaacgatgc-3’	This study
VNL025	qRT-PCR OCN008 and BAA-450 vcpA reverse	5’-ctcgtttcacttcagcgaa-3’	This study
VNL026	qRT-PCR OCN008 and BAA-450 vcpB forward	5’-atgaaaatagccaagcgttttt-3’	This study
VNL027	qRT-PCR OCN008 and BAA-450 vcpB reverse	5’-agttgattggtttgcttaaataaact-3’	This study
VNL070	Amplify gene locus OCN008 recA forward	5’-atgaacaaatcggagaaagtgatgg-3’	This study
VNL071	Amplify gene locus OCN008 recA reverse	5’-ttatagctcttcttgctctggcatt-3’	This study
VNL072	Amplify gene locus BAA-450 recA forward	5’-ttgattgcctcagaggactctgg-3’	This study
VNL073	Amplify gene locus BAA-450 recA reverse	5’-ttatagctcttcttgctctggcatt-3’	This study

References

1. Ushijima B, Videau P, Burger AH, Shore-Maggio A, Runyon CM, Sudek M, Aeby GS, Callahan SM. 2014. Vibrio coralliilyticus Strain OCN008 Is an Etiological Agent of Acute Montipora White Syndrome. Appl Environ Microbiol 80:2102–2109.
2. Ushijima B, Videau P, Poscablo D, Vine V, Salcedo M, Aeby G, Callahan SM. 2014. Complete genome sequence of Vibrio coralliilyticus strain OCN014, isolated from a diseased coral at

Palmyra Atoll. Genome Announc 2:8–9.

3. Bassler BL, Greenberg EP, Stevens AM. 1997. Cross-species induction of luminescence in the quorum-sensing bacterium Vibrio harveyi. J Bacteriol 179:4043–4045.
4. Guillemette R, Ushijima B, Jalan M, Häse CC, Azam F. 2020. Insight into the resilience and susceptibility of marine bacteria to T6SS attack by Vibrio cholerae and Vibrio coralliilyticus. PLoS One 15:1–19.
5. Waters CM, Bassler BL. 2006. The Vibrio harveyi quorum-sensing system uses shared regulatory components to discriminate between multiple autoinducers. Genes Dev 20:2754–2767.
6. Simpson CA, Podicheti R, Rusch DB, Dalia AB, van Kessel JC. 2019. Diversity in natural transformation frequencies and regulation across vibrio species. MBio 10:1–16.
7. Simpson CA, Petersen BD, Haas NW, Geyman LJ, Lee AH, Podicheti R, Pepin R, Brown LC, Rusch DB, Manzella MP, Papenfort K, van Kessel JC. 2021. The quorum-sensing systems of Vibrio campbellii DS40M4 and BB120 are genetically and functionally distinct. Environ Microbiol 23:5412–5432.
8. Mass S, Cohen H, Podicheti R, Rusch DB, Gerlic M, Ushijima B, van Kessel JC, Bosis E, Salomon D. 2024. The coral pathogen Vibrio coralliilyticus uses a T6SS to secrete a group of novel anti-eukaryotic effectors that contribute to virulence. PLoS Biol 22:e3002734.
9. Le Roux F, Binesse J, Saulnier D, Mazel D. 2007. Construction of a Vibrio splendidus mutant lacking the metalloprotease gene vsm by use of a novel counterselectable suicide vector. Appl Environ Microbiol 73:777–784.
